# Synthesising the existing evidence for non-pharmacological interventions targeting outcomes relevant to young people with ADHD in the school setting: systematic review protocol

**DOI:** 10.1186/s13643-022-01902-x

**Published:** 2022-02-16

**Authors:** Abigail E. Russell, Darren Moore, Amy Sanders, Barnaby Dunn, Rachel Hayes, Judi Kidger, Edmund Sonuga-Barke, Linda Pfiffner, Tamsin Ford

**Affiliations:** 1 University of Exeter College of Medicine and Health, South Cloisters, St Luke’s Campus, Exeter, EX1 2LU UK; 2 University of Bristol, Bristol, UK; 3 Kings College London, London, UK; 4 University of California San Francisco, San Francisco, USA; 5 University of Cambridge, Cambridge, USA

**Keywords:** Systematic review, ADHD, School, Non-pharmacological interventions, Self-esteem, Executive functioning, School relationships, Intervention mapping, Organisation skills, Quality of life, Global functioning

## Abstract

**Background:**

Children and adolescents with attention-deficit/hyperactivity disorder (ADHD) have impairing levels of difficulty paying attention, impulsive behaviour and/or hyperactivity. ADHD causes extensive difficulties for young people at school, and as a result these children are at high risk for a wide range of poor outcomes. We ultimately aim to develop a flexible, modular ‘toolkit’ of evidence-based strategies that can be delivered by primary school staff to improve the school environment and experience for children with ADHD; the purpose of this review is to identify and quantify the evidence-base for potential intervention components. This protocol sets out our plans to systematically identify non-pharmacological interventions that target outcomes that have been reported to be of importance to key stakeholders (ADHD symptoms, organisation skills, executive-global- and classroom-functioning, quality of life, self-esteem and conflict with teachers and peers). We plan to link promising individual intervention components to measured outcomes, and synthesise the evidence of effectiveness for each outcome.

**Methods:**

A systematic search for studies published from the year 2000 that target the outcomes of interest in children and young people aged 3–12 will be conducted. Titles and abstracts will be screened using prioritisation software, and then full texts of potentially eligible studies will be screened. Systematic reviews, RCTs, non-randomised and case-series studies are eligible designs. Synthesis will vary by the type of evidence available, potentially including a review of reviews, meta-analysis and narrative synthesis. Heterogeneity of studies meta-analysed will be assessed, along with publication bias. Intervention mapping will be applied to understand potential behaviour change mechanisms for promising intervention components.

**Discussion:**

This review will highlight interventions that appear to effectively ameliorate negative outcomes that are of importance for people with ADHD, parents, school staff and experts. Components of intervention design and features that are associated with effective change in the outcome will be delineated and used to inform the development of a ‘toolkit’ of non-pharmacological strategies that school staff can use to improve the primary school experience for children with ADHD.

**Trial registration:**

PROSPERO number CRD42021233924

**Supplementary Information:**

The online version contains supplementary material available at 10.1186/s13643-022-01902-x.

## Background

Children and adolescents with attention deficit/hyperactivity disorder (ADHD) have impairing levels of difficulty paying attention, impulsive behaviour or hyperactivity [1, 2]. The core symptoms of ADHD are dimensional traits, so those with high levels of symptoms not meeting clinical diagnostic thresholds also experience similar difficulties [3, 4]. The prevalence of ADHD in children and adolescents is 2–7% [5]. ADHD causes extensive impairment for young people at school as well as home, and as a result these children are at high risk for poor academic attainment, other physical and mental health conditions and unintentional injuries [6–8]. Unfortunately, many young people with persistent ADHD find themselves in trouble with the police [4], and struggle to maintain friendships and relationships [6]. In spite of these difficulties and impairments, each young person with ADHD is a unique individual with their own strengths. Emerging evidence suggests that individuals with ADHD may be very creative, or able to ‘hyper-focus’ on things that engage their interest [9, 10]. They may think outside the box, and bring energy and fun to group situations. Empirical research into the strengths of children with ADHD is lacking and findings are mixed, although associations have been noted between ADHD and entrepreneurship [11].

The school environment poses some extreme challenges for children with ADHD [12, 13]. Having to stay still and quiet in classroom scenarios can be extremely difficult, focussing on work for long periods of time may be frustrating and tiring, and transitions between lessons or breaks can cause anxiety and exacerbate forgetfulness. Teacher-student relationships are often poorer for children with ADHD than their typically developing peers, which puts children with ADHD at risk for social exclusion [14, 15]. This, alongside perceptions that the young person can control their behaviour, yet is choosing not to, may lead to stigma and low self-esteem [13, 14]. Shouting out in class and negative interactions with teachers, coupled with difficulty expressing emotions in an age-appropriate manner, can lead to repeated exclusions or expulsion from school; children with ADHD experience higher rates of school exclusions [16]. Academic difficulties and co-occurring conditions may lead to placement in special educational provision, or home tutoring [8, 12]. Effective interventions are therefore needed to support these children through school.

Although there are pharmacological treatments for ADHD, these are not acceptable or tolerable for all children, and tolerance may develop after a few years [17]. Even with medication, children with ADHD do not do as well as those without ADHD in terms of both health- and school-based outcomes [18], and other options for school-based support are required that account for the complexity of successfully managing ADHD. There are often long waits for assessment and diagnosis of ADHD which impacts on the support a child receives in school; a recent survey reported the mean time from parents noticing symptoms to receiving a diagnosis of 31.9 months, with an 18.3 month average wait from first raising concerns with a doctor to receiving a diagnosis [19]. Although medication may acutely alleviate symptoms, support with skill development and other aspects of behaviour are still needed. Many studies have attempted to improve outcomes for children with ADHD in the school setting (e.g. [20–22]). In spite of this, the evidence for which parts of these interventions confer effectiveness is mixed. Non-pharmacological interventions are not always focussed on individual child deficits; they can also address the wider environment around the child to improve support [23]. These interventions may therefore benefit other children beyond the individual with ADHD.

We ultimately aim to develop a flexible, modular ‘toolkit’ of evidence-based strategies that can be delivered by primary (i.e. attended by children ages 5–11 years) school staff to improve the school environment and experience for children with ADHD; the purpose of this review is to identify and quantify the evidence-base for components of non-pharmacological interventions, and explicitly consider theoretical mechanisms of behaviour change within the promising components, in order to select strategies for the toolkit. Many existing school-based interventions for ADHD are complex and multi-component, targeting multiple outcomes in each child regardless of individual strengths or weaknesses [24]. Due to this, it is difficult to disentangle which components of these interventions or characteristics of individual participants are associated with effectiveness and methods such as intervention mapping are needed to understand this. In 2018, a qualitative comparative analysis was conducted to attempt to answer this question; findings indicated interventions targeting self-regulation and those delivered 1:1 may be associated with improvements in academic outcomes [24]. Further work is still needed to ascertain the best evidence for improving other outcomes for children with ADHD in the school environment given the complexity highlighted in existing reviews. For example, in Richardson et al.’s series of reviews, shorter interventions were more effective which appears paradoxical; however, in qualitative studies, teachers have reported having little time to implement interventions, and in practice report using brief, flexible strategies tailored to the individual child or environment [23, 25, 26]. It may be that longer, complex interventions with many components were implemented with poor fidelity by school staff in existing studies due to busy classrooms and competing demands. There is also currently a poor understanding of mechanisms of change of individual intervention components (and therefore the reasons that intervention components may or may not work), and a lack of explicit consideration of developmental stage and context [27]. Furthermore, existing interventions are poorly implemented (likely due to their complexity) when school staff report using brief varied strategies flexibly in practice [26].

Existing studies of school-based interventions for ADHD have measured over 50 different outcomes [24]. Which of these outcomes were of most importance to people with ADHD and other key stakeholders have been recently identified through a Delphi survey. Consensus was obtained on the outcomes or targets that were considered to be most important to people with ADHD (including children), parents, school staff and ADHD professionals (in the context of developing and delivering a school-based intervention) [28]. These broad categories of outcomes inform and set the parameters of the current review. The current review will also synthesise and extend existing reviews of non-pharmacological and school-based interventions for ADHD (e.g. [27, 29–32]).

In this review, we aim to systematically identify non-pharmacological interventions that target the above outcomes of importance to people with ADHD, parents, school staff and ADHD experts. The aim is to link individual intervention components to measured outcomes using quantitative synthesis and then to apply methods from intervention mapping to understand hypothesised processes of behaviour change. This review aims to synthesise the evidence of effectiveness of both whole interventions and intervention components (where possible) for each outcome. The review will not be restricted to only those with confirmed ADHD; rather we will seek to identify evidence-based intervention components that may be applicable to those with traits of ADHD and could be translated to the school context. Therefore, the population of primary interest is children and young people age 3–12, the intervention is any non-pharmacological intervention where one of the above outcomes is measured, and the comparator is no intervention or treatment as usual (including within-subject control).

### Aims and research questions

The aim of this review is to systematically identify non-pharmacological interventions that target outcomes of importance to people with ADHD, parents, school staff and ADHD experts (listed in Table 1). We further aim to synthesise the evidence regarding which interventions and components of interventions are associated with improvements in the target outcomes. Therefore there are two main research questions that will be answered with different evidence synthesis strategies:

**Table 1:** The most important outcomes for a school-based ADHD intervention to target, consensus reached by Delphi survey

Outcome	
Inattention	
Hyperactivity	
Impulsivity	
Conflict with teachers and peers	
Organisational skills	
Self-esteem	
Classroom functioning	
Executive functioning	
Global functioning/quality of life	

#### Research question 1

To what extent do non-pharmacological interventions for children and adolescents improve outcomes that have been established as being important to people with ADHD, parents and school staff, and what components of interventions appear to be associated with effectiveness?

#### Research question 2

Why are the components identified in research question 1 effective at ameliorating ADHD symptoms or associated outcomes and what are the mechanisms of behaviour change involved?

## Methods

A PRISMA-P checklist is included in the Additional file 1, and this protocol is registered on Prospero (ref CRD42021233924).

### Eligibility criteria

#### Population

Children and young people age 3–12 where ADHD or any of the core symptoms (impulsivity, inattention or hyperactivity) are mentioned in the title or abstract. Studies solely including “typically developing” populations will be excluded, as will studies of populations with acquired brain injury.

#### Intervention

Any non-pharmacological intervention or strategy, for example interventions or strategies may be psychological, cognitive, behavioural, or aim to change the learning environment. Interventions that intervene with diet or nutrition (including dietary supplements) will be excluded. Interventions requiring specialist equipment not commonly available to schools (e.g. EEG or specialist animals such as horses) will be excluded. Studies will be excluded if the intervention clearly requires a trained therapist to deliver it. Studies will not be excluded if young people are taking medication (for ADHD or other conditions). Studies that report solely on parent training, with no child-delivered component or school-relevant outcomes will be excluded, as will studies of the overarching effect of “summer treatment programmes” as these are intensive extended intervention programmes. A separate review of summer treatment programmes for ADHD is planned.

#### Comparator

For group designs, the comparator will be no intervention or treatment as usual (including medication). For within-subject designs, the comparator will be each participants’ baseline data.

#### Outcomes

Eligible studies will provide pre- and post- measures that capture a facet of at least one of the following child-focussed outcomes. Some outcomes may overlap, for example planning is an executive function, but also an organisational skill. Outcome measures may be recorded by parents, teachers, the young person or through observational measures or computer-assisted technology. We will include studies of both (i) interventions that directly target the outcomes, and (ii) studies that measure any of these outcomes although they were not the explicit focus of the intervention.

1. Hyperactivity
2. Inattention (and attention but not “joint attention” referring to eye-gaze in autism studies)
3. Impulsivity
4. Organisational skills, including organised actions, time management and task planning [33]
5. Teacher conflict—or relationships with teachers
6. Peer conflict—or relationships with peers, and other indicators of social impairment such as friendships, peer relationships and bullying
7. Self-esteem
8. Executive functioning, including working memory, inhibitory control, shifting between tasks or cognitive flexibility, emotional self-regulation, initiating, planning and problem solving, self-monitoring [34]
9. Classroom functioning, e.g. task-relevant behaviours, communication, engagement, social skills, leadership skills, learning problems, problem behaviours, academic competence, classroom physical environment [35, 36]
10. Global functioning and quality of life

### Types of study to be included

For research question 1, eligible studies will report changes in any of the outcomes listed above following non-pharmacological intervention and include control or comparison data. Systematic reviews that include studies meeting the above criteria, randomised and non-randomised controlled trials will be included for the quantitative synthesis. In addition to these study designs, controlled before and after studies, case-control studies or case series (including multiple baseline designs) are eligible study designs for research question 2. Individual study participants can be their own controls, e.g. using pre-intervention baseline data. Studies that include one intervention group that has the same measure pre- and post-treatment will be eligible for inclusion, as long as there are data available for a comparative ‘control’ period pre-treatment. Qualitative studies and individual case studies will be excluded from the synthesis, although they may inform the intervention mapping analysis. Process evaluations will be eligible study designs for research question 2 where they report on an intervention study that meets inclusion criteria. Where there are existing high quality systematic reviews that match our inclusion criteria, we will include these reviews and only synthesise primary research studies published since the review search dates. It is anticipated that most existing systematic reviews will not match our aims precisely, in this case individual eligible studies within reviews will be included.

Given the ultimate aim of extracting detailed intervention information and plans to request intervention manuals from authors, we have made the pragmatic decision to include only studies published in English from 2000 to the search date (late 2020). This is based on experience from prior efforts to contact authors of school-based ADHD interventions; authors publishing more than two decades ago were often not traceable, or reported no longer having intervention manuals or further information. Prior to final analysis, searches will be re-run to identify any further recent publications. Conference abstracts will be excluded, and dissertations will be included. Forward and backward citation searching of included articles will also be conducted.

### Context

Although the ultimate aim is to translate evidence to a school setting, eligible studies can be in any setting (home, school, after-school, hospital school, or clinical settings). As part of the scoping of literature, searches and title and abstract screening will include children and adolescents up to the age of 18; however, given the volume of eligible literature and that studies conducted with older age groups may have limited translatability to primary school settings, full-text screening will include only studies of children aged 3–12 years as the eligible study population. Following full-text screening, if there are insufficient studies in this younger population (*n* < 3 for any of the outcomes listed above), full texts will be re-screened and studies of secondary school pupils (age 12–18 years) will be included. Depending on the volume of evidence available for each outcome, studies based in the school setting will be prioritised over those in other settings.

### Search strategy

The following databases and registries will be searched: Medline, PsycINFO, Australian Education Index, ERIC, Education research complete, British Education Index, Embase, Health Management Information Consortium, Social Policy and Practice (via OvidSP); ASSIA, Social Sciences Citation Index, Conference Proceedings Citation Index; Conference Proceedings Citation Index–Social Science and Humanities.

In addition, the following databases will be screened for relevant studies: The Cochrane Library, Cochrane Database of Systematic Reviews (CDSR), Database of Abstracts of Reviews of Effects (DARE), Cochrane Central Register of Controlled Trials (CEN-TRAL), Cochrane Methodology Register (CMR), Health Technology Assessment (HTA) and The Campbell Library ISRCTN Registry. An example draft search strategy for PsycINFO can be seen in Table 2. Searches will have two steps, the first step will include *ADHD terms*, AND *at least one of the other outcome terms* (combined with OR), AND *intervention terms*. The second search will include *ADHD terms* AND *intervention terms* and exclude those results already present from step 1. This second step will capture studies that measure only the ADHD-specific symptom outcomes but none of our other outcomes of interest. Records from the two steps will be combined to form the search results. Scoping searches revealed that these terms are very broad: to focus the screening, additional NOT terms were applied to the records following all database searches (see Table 3).

**Table 2:** Example search strategy, PsycINFO

**ADHD terms**	
attention deficit disorder with hyperactivity	
ADHD	
attention deficit/hyperactivity disorder	
ADD	
attention deficit	
hyperactiv*	
hyperk*	
inattent*	
impulsiv*	
restless*	
overactiv*	
(attention (problem or difficult* or disorder* or issue))	
**Organisation skills terms**	
organi?ation* skill*	
time management	
planning	
preparing material*	
task management	
homework	
((self or personal) adj2 organis*)	
**Teacher and peer relationship terms**	
teacher relationship*	
peer relationship*	
friend*	
teacher conflict	
peer conflict	
classroom conflict	
pupil relationship*	
student-teacher	
pupil-teacher	
peer-peer	
teacher pupil relationship*	
teacher student relationship*	
(Family adj2 school adj (partnership* or relationship* or involvement))	
(Parent adj2 school adj (partnership* or relationship* or involvement))	
(school adj2 parent adj (partnership* or relationship* or involvement))	
(home adj2 school adj (partnership* or relationship* or involvement))	
**Self-esteem terms**	
self esteem	
self-esteem	
self-worth	
self worth	
value	
self-respect	
self respect	
Self-confiden*	
**Executive functioning terms**	
working memory	
inhibit*	
task shift*	
cognitive flexib*	
emotional regulat*	
self-regulat*	
planning	
problem solving	
self-monitor*	
**Classroom functioning terms**	
engagement*	
classroom funct*	
social skill*	
leadership*	
academic*	
attainment	
classroom environment*	
learning environment	
learning establishment	
teaching environment	
**Global functioning and quality of life terms**	
global function*	
quality of life	
QoL	
happiness	
satisfaction	
assessment of function*	
social function*	
psychological function*	
global impairment*	
**Intervention terms**	
intervention*	
strateg*	
program*	
project*	
train*	
support*	
therap*	
treatment	
(Behavio?r* adj2 (management or modification* or medicine or treatment*))	
(education* adj2 (management or modification* or treatment*))	
(classroom adj2 (management or modification* or treatment*))	
(playground adj2 (management or modification*))	
(psychosocial adj2 (management or modification* or treatment*))	
(cognitive adj2 (management or modification* or treatment*))	
behavio?r change technique*	
mentor*	
counsell*	
coach*	
social skills	
social problem solving	
life skills	
CBT	
cognitive behavio?r*	
cognitive therap*	
**Not terms**	
TBI or (traumatic brain injury)	
University*	
higher education*	

Each category of terms combined with ‘or’, then final search is:

Search 1; (ADHD terms) AND (organisation skills terms) OR (teacher and peer relationship terms) OR (self-esteem terms) OR (executive functioning terms) OR (classroom functioning terms) OR (global functioning and QoL terms) AND (intervention terms) NOT (not terms), restricted to year=2000/2020; Search 2; (ADHD terms) and (intervention terms) not (not terms or results from search 1), restricted to year=2000/2020 * is a wildcard symbol used to denote any characters following the word stem

**Table 3:** List of additional “not” terms

Term	
Cell induced	
Protein	
Rat	
Cancer	
Tumo?r	
Inhibitor	
Cocaine	
Immune	
Covid	
Infection	
Autoimmune	
Inflammatory	
Gene	
Mutat*	
Genetic variants	
Bladder	
Oab	
Mirabegron	
Solifenacin	
Incontinen*	
Kinase	
Alpha	
Phosphorylate*	
Receptor	
Binding	
Nucleus	
Stroke	
Renal	
Hyperalaemia	
Kidney	
Ckd	
Platelet	
Complement	
Serum	
Blood	
Biomarker	
Plasma	
Lesion	
Hyperkerat*	
Psoriasis	
Cutaneous	
Keratosis	
Aky	
Mapk	
Phosphor*	
Diabet*	
Insulin	
Metabolic	
Glucose	
Mellitus	
Neuronal	
Alzheime*	
Neuron	
Microglia	
Urinary	
Detrusor	
Voiding	
Urodynamic	
Calcium	
Hypertension	
Cardiovascular	
Carotid	
Arterial	
Myocardial	
Oxidative	
Autophagy*	
Botulinum	
Intravesical	
Neurogenic	
Postoperative	
Surgical	
Preoperative	
Prolapse	
Hyperkyphosis	
Kyphosis	
Thoracic	
Delirium	
Dementia	
Icu	
Methylation	
Epigenetic	
Histone	
Hepatic	
Thyroid	
Testosterone	
Adrenal	
Antibodies	
Ovarian	
Serum	
Hypothyroidism	
Prostate	
Prostatic	
Osteockast*	
Enzyme	
Sperm	
Progesterone	
Fertility	
Semen	
“Adults with ADHD”	
“Adult ADHD”	

Additional search strategies include forward and back citation chasing, contacting experts and searching through key journals (where journals or authors are highly recurrent in included studies). Potentially eligible trials identified through trial registrations will be followed-up to identify relevant publications, and where trials appear ongoing authors will be contacted to enquire if they have relevant data to contribute.

### Study selection

Two reviewers will screen titles and abstracts, and then full-text records to assess study eligibility. Disagreements will be resolved through discussion, with a third reviewer (DM) if necessary. Screening and search records will be kept following a PRISMA diagram, with reasons recorded for studies that are excluded at full-text screening. Because scoping searches indicated a very high number of records would be retrieved from database searches (i.e. > 130,000), title and abstract screening will utilise *Swift-Review* software to minimise burden and facilitate the screening process [37]. This uses a ‘literature prioritisation’ algorithm, text mining and machine learning in order to prioritise screening for abstracts that appear eligible based on decisions made thus far. Piloting will involve reviewers independently making decisions on 100 titles and abstracts (that include eligible studies measuring the full range of outcomes of interest and studies that are ineligible). Reviewers will then screen from the top of the prioritised list, and the library will be re-prioritised frequently throughout screening to enhance the prioritisation algorithm (approximately every 100 records). Title and abstract screening will continue until 100 consecutive records are ineligible, and then after reprioritisation, a further 100 are not eligible. A random 100 records from those remaining will then be screened to ensure all are ineligible prior to concluding title and abstract screening.

Full-text screening will be conducted by three reviewers. All reviewers will screen 10% of the records to assess concordance. Providing reviewers have a high level of agreement (> 95%) on studies meeting inclusion criteria, the remaining full texts will be each screened by one individual. If not, double-screening will continue until concordance is reached. Screening will result in a series of studies meeting inclusion criteria across all outcomes.

Following screening, additional searches will be conducted and authors contacted in order to identify additional data relating to the implementation of included interventions, for example process evaluations, intervention manuals and qualitative analyses.

### Data extraction

A data extraction form will be developed and piloted with 10 studies from a range of designs. A separate data extraction form will be used for systematic reviews. Data will be extracted by one reviewer, with data from the first 10% of included studies extracted and checked by a second reviewer. The second reviewer will also scrutinise extraction of data used for calculating effect sizes, intervention descriptions, and categorisation of outcome measures for at least an additional 10% of studies, as these are considered aspects of data extraction where accuracy is critical. If discrepancies are identified, further studies will be double-extracted until consistency is reached.

For individual intervention studies (including eligible studies within systematic reviews), descriptive data on the study sample will be extracted (number of participants, age, sex, clinical characteristics), along with detail of the study year, author, funding source, country, study design, setting, intervention and control group conditions. As much detail as possible will be extracted regarding the nature, content and implementation of the intervention, following the *template for intervention description and replication* (TIDieR) for describing interventions, which include items on reporting fidelity and implementation [38]. Information will be extracting regarding who delivers the intervention and formats of delivery, dosage and duration of baseline, intervention and follow-up periods. As much detail as possible will be extracted relating to theoretical approaches used, implied or explicitly referred to. Where participants represent a selected group (e.g. those with ADHD), information on diagnostic assessment and current treatments will be extracted where these data are available.

Components of interventions in each study will be paired with the estimate of effect on the outcomes of interest. These are likely to be challenging to disentangle, and for this aspect of the analysis the identified intervention components will be paired with any pertinent outcomes measured. For example, if an intervention includes four components and two relevant outcomes, the effect size estimate will be recorded for each pairing of component and outcome; eight effect sizes in this example (of which the four effect estimates for each outcome will be the same). It is acknowledged that this approach will be somewhat reductionist where interventions are poorly described; however, synthesising these effects across studies should allow for identification of promising components of interventions, to be further evaluated through intervention mapping.

For each intervention-outcome pairing, participant numbers, means and standard deviations (SDs) for outcome measures will be extracted, or a measure of intervention effect with an estimate of precision such as confidence intervals. These will be converted to *Hedge’s g*, a standardised measure of study effect size that corrects for overestimation of the true population effect [39]. For non-randomised studies of interventions, we will follow Cochrane guidance and extract estimates of effect and precision as well as information about how the effect estimate was derived (e.g. the confounders controlled for) [40]. For high-quality systematic reviews that fully meet our inclusion criteria, similar data will be extracted including intervention descriptions in included studies, as well as lists of included studies.

### Risk of bias (quality assessment)

There are multiple tools that can be used to assess risk of bias. The Joanna Brigg’s Institute tools for critical appraisal will be utilised because it has separate quality appraisal tools available for appraisal of systematic reviews, RCTs and non-randomised experimental studies [41]. There are no guidelines of which we are aware to appraise the quality of interventions and implementation, so to appraise the quality of interventions, and the quality of implementation of the intervention, we will develop an appraisal tool that reflects the components of the TiDieR checklist and best-practice recommendations on assessing implementation and fidelity [38].

### Strategy for data synthesis

Data will be synthesised separately for the two research questions. For research question 1, eligible study designs will be systematic reviews, randomised and non-randomised trials (i.e. between-group studies). For research question 2, within-group studies such as case-series and multiple baseline studies will also be eligible for inclusion, along with process evaluations and intervention manuals.

Screening will result in a series of studies meeting inclusion criteria for each of the outcomes. The evidence for each outcome will be synthesised separately, where constructs overlap substantially these will be merged and discussed as pertinent to both outcomes.

Depending on the level of evidence and volume of research available for each outcome, data synthesis will vary. Should there be a large number of eligible systematic reviews, research question 1 may include a synthesis of reviews or a ‘review of reviews’ in addition to the analysis linking components of primary studies to intervention effect estimates; we will use the AMSTAR tool to assess review quality and follow best-practice guidance [42]. For systematic reviews that meet inclusion criteria, we will conduct a separate synthesis to explore whether primary research that has been published since the review date fits the reported findings. Data from interventions included within systematic reviews will be extracted and synthesised in addition. Where it is not possible to include a systematic review, for example if many of the studies in the review are not pertinent to the research questions, individual study details will be extracted from reviews and checked for eligibility.

Where included studies are sufficiently homogeneous, random-effects meta-analysis will be used to assess the cumulative evidence for intervention effectiveness. Heterogeneity will be assessed using the *I*^2^ statistic [43], and funnel plots and Egger’s regression will be used to investigate potential effects of publication bias [44]. We will prioritise quantitative analysis of existing meta-analyses and randomised controlled trials for each outcome, and conduct meta-analysis or quantitative synthesis of other study designs where appropriate, when sufficient systematic reviews (at least 1) or RCTs (at least 5 without a high heterogeneity estimate) are unavailable. Moving beyond the component-outcome analysis, subgroup analyses and meta-regression will be used to examine the influence of specific intervention or study features associated with effectiveness, for example by examining the impact of setting, duration, delivery agent, or type of intervention, the population studied, the country and region of the study setting and the gender of participants on outcomes. The composition and nature of control conditions such as “treatment as usual” will vary depending on the location and design of included studies, and we intend to report a descriptive table detailing clinical guidance for treatment of ADHD for the countries in which included studies are set.

Given that interventions and measures for the pre-school aged population may differ from those who are of school age, we will also assess whether the effectiveness of non-pharmacological interventions varies by the age of the study population. The strength of the body of evidence for each outcome in research question 1 will be assessed using GRADE [45].

Synthesis of non-randomised group-based study designs can be complex; these studies are subject to a higher risk of bias than randomised designs [46], and the direction of bias between studies may vary due to different features of study design [40]. However, evidence from non-randomised studies will complement evidence from RCTs and may be the best available evidence for some outcomes, and so if the study designs and measures are sufficiently homogeneous, meta-analyses and meta-regression will be undertaken. Findings from within-subject study designs where each participant provides their own control data will be used to inform the intervention mapping analysis for research question 2.

The findings from the data synthesis will identify and quantify the current evidence relating to whether outcomes of interest can be affected by non-pharmacological interventions for children and young people, and begin to identify broad features of interventions that are associated with effectiveness. These findings will be discussed with a stakeholder group comprised of people with ADHD, parents, school staff and experts in education and psychology in order to discuss the context and implications of the results, and the feasibility of delivering these in primary schools.

Given the anticipated difficulty of establishing whether individual intervention components of multi-component complex interventions have an effect on outcomes, following the initial evidence synthesis to quantify effect sizes, intervention mapping [47] will be applied to the most promising strategies with the strongest evidence for effectiveness, and studies meeting inclusion criteria that report using these components will be scrutinised. Intervention mapping is a theory- and evidence-based approach to understanding which components of interventions are likely to result in behaviour change, so the theoretical pathway linking each intervention component with the outcome will be elicited. Logic models of behaviour change will be constructed for each promising intervention-outcome component, underpinned by evidence and theory, and components that align with empirical and theoretical evidence will be selected for inclusion in the toolkit [47]. In order to achieve this, intervention manuals, process evaluations and other publications relating to interventions will also be retrieved by contacting authors or via searches, findings and implications from relevant systematic reviews will be integrated, and literature relating to identified theories such as behaviour change and complex systems theories will also be drawn upon.

## Discussion

This systematic review will identify and quantify the evidence for non-pharmacological interventions that target a range of outcomes of importance to people with ADHD, parents, school staff and experts. These include the core symptoms of ADHD, as well as relationships or conflict with staff and peers (including indicators of social impairment), self-esteem, organisation skills and a variety of aspects of functioning. This evidence synthesis will move beyond the contributions of existing studies in this field by providing up-to-date quantitative assessments of effectiveness, and explicitly mapping the evidence for individual intervention components to change the outcomes of interest. We will also synthesise and extend existing systematic reviews in these areas by identifying subsequent studies and synthesising findings in light of what is already known. Intervention mapping will extend the evidence synthesis by integrating theory and evidence for the most promising intervention components, and explicitly mapping out pathways to behaviour change. This will be used to create logic models of behaviour change that would be required for each component to be effective, ultimately facilitating the translation of identified evidence-based components and strategies into a toolkit for non-specialists to use working in schools with children with traits of ADHD.

## Supplementary Information

**Additional file 1.** PRISMA-P 2015 Checklist.

## Data Availability

N/A

## Abbreviations

ADHD: Attention deficit/hyperactivity disorder
RCT: Randomised controlled trial
TiDieR: Template for intervention description and replication
SD: Standard deviation
